# Challenges in Diagnosis and Therapeutic Approach of Acute on Chronic Liver Failure—A Review of Current Evidence

**DOI:** 10.3390/biomedicines11071840

**Published:** 2023-06-26

**Authors:** Cristina Maria Marginean, Denisa Pirscoveanu, Mihaela Popescu, Corina Maria Vasile, Anca Oana Docea, Radu Mitruț, Iulia Cristina Mărginean, George Alexandru Iacob, Dan Mihai Firu, Paul Mitruț

**Affiliations:** 1 Department of Internal Medicine, University of Medicine and Pharmacy of Craiova, 200349 Craiova, Romania; 2 Department of Neurology, University of Medicine and Pharmacy of Craiova, 200349 Craiova, Romania; 3 Department of Endocrinology, University of Medicine and Pharmacy of Craiova, 200349 Craiova, Romania; 4 Department of Pediatric Cardiology, “Marie Curie” Emergency Children’s Hospital, 041451 Bucharest, Romania; 5 Department of Toxicology, University of Medicine and Pharmacy of Craiova, 200349 Craiova, Romania; 6 Department of Cardiology, University and Emergency Hospital, 050098 Bucharest, Romania; 7 Faculty of Medicine, University of Medicine and Pharmacy of Craiova, 200349 Craiova, Romania; 8 Ph.D. School Department, University of Medicine and Pharmacy of Craiova, 200349 Craiova, Romania

**Keywords:** acute-on-chronic liver failure, liver cirrhosis, gut microbiome, liver transplantation, organ failure

## Abstract

Acute-on-chronic liver failure (ACLF) is a syndrome characterized by acute and severe decompensation of chronic liver disease (CLD) correlated with multiple organ failure, poor prognosis, and increased mortality. In 40–50% of ACLF cases, the trigger is not recognized; for many of these patients, bacterial translocation associated with systemic inflammation is thought to be the determining factor; in the other 50% of patients, sepsis, alcohol consumption, and reactivation of chronic viral hepatitis are the most frequently described trigger factors. Other conditions considered precipitating factors are less common, including acute alcoholic hepatitis, major surgery, TIPS insertion, or inadequate paracentesis without albumin substitution. Host response is likely the primary factor predicting ACLF severity and prognosis, the host immune response having a particular significance in this syndrome, together with the inflammatory cascade. The management of ACLF includes both the prevention of the precipitating factors that lead to acute liver decompensation and the support of vital functions, the prevention and management of complications, the estimation of prognosis, and the opportunity for liver transplantation.

## 1. Introduction

Acute-on-chronic liver failure (ACLF) represents a condition characterized by acute decompensation of underlying chronic liver disease (CLD) correlated with multiple organ failure, poor prognosis, and increased mortality.

The EASL (European Association for the Study of the Liver), NACSELD (North American Consortium for the Study of End-Stage Liver Disease), and APASL (Asia Pacific Association for the Study of the Liver) have proposed different definitions of ACLF syndrome; all are based on clinical features, associated with alterations of biological liver tests combined with organ failure [1,2].

More than 13 distinct definitions of ACLF have been advanced so far, but the definitions from APASL and the European Association for the Study of Chronic Liver Failure (EASL-CLIF) Consortium are universally recognized [3,4].

Thus, the consensus of the Asia-Pacific Association for the Study of the Liver (APASL) defined ACLF as acute liver injury manifested by jaundice (total bilirubin ≥ 5 mg/dL) and coagulopathy (INR ≥ 1.5), which evolves within four weeks with ascites with/without encephalopathy in a patient previously diagnosed with CLD.

According to the EASL-CLIF criteria, ACLF emerges in patients with previously compensated or decompensated liver cirrhosis who develop other organ failures. The EASL-CLIF criteria exclude patients previously diagnosed with HCC (hepatocellular carcinoma), HIV (Human Immunodeficiency Virus), or severe comorbidities [5]. The EASL-CLIF criteria, compared to the NACLSELD and APASL criteria, define a relatively enlarged spectrum of disease severities, including ACLF disease phases with better prognosis (phases 1/2) and outcomes, as well as advanced phases with multiple organ failure, high short-term mortality, and poor prognosis [4,6].

The NACSELD criteria were defined initially for patients hospitalized with decompensated cirrhosis and bacterial infections, excluding previous malignancies, HIV, or previous organ transplantation [5]. These criteria completed the EASL-CLIF criteria; therefore, ACLF was defined by the presence of at least two extrahepatic organ failures, including shock, the need for vasopressors despite adequate intravenous fluid therapy, encephalopathy stage 3/4, renal replacement, and/or mechanical ventilation [1]. The NACSELD definition does not differentiate between distinct disease phases and stages of ACLF, so it may underdiagnose ACLF.

The APASL criteria used a wide disease spectrum focusing on liver failure as central to the ACLF definition [2]. The APASL and ACLF definitions were derived from acute liver failure, which develops in patients with preexisting chronic liver disease or liver cirrhosis, but these definitions exclude patients with decompensated cirrhosis or a history of infection [3,5]. Therefore, patients with acute hepatic injury, such as reactivation of viral hepatitis, drug-induced liver injury, or surgical procedures, and those with cirrhosis without any previous infections or variceal hemorrhage meet the APASL ACLF criteria if these factors can lead to jaundice and coagulopathy and are followed by ascites or hepatic encephalopathy within four weeks. Although the APASL definition presumes the existence of underlying chronic liver disease, unlike the EASL-CLIF criteria, it does not distinguish between ACLF with or without cirrhosis [5,7].

Despite the worldwide diversity of ACLF definitions related to diagnosis, precipitating factors, underlying comorbidities, organ failure, and management, patients with ACLF have an invariably poor prognosis. The most recently and exhaustive guideline for the current data in ACLF was elaborated in 2022 by Bajaj et al., and it represents the official practice recommendation of the American College of Gastroenterology, being structured on statements considered useful and clinically relevant. The guideline provides a list of recommendations regarding definitions, diagnosis, key pathogenetic mechanisms, precipitating factors, and a complex therapeutic approach [5].

## 2. Etiological Factors of ACLF

In 40–50% of ACLF cases, the trigger has not been recognized. For many of these patients, bacterial translocation and systemic inflammation are thought to be the determining factors [8]; in the other 50% of patients, sepsis, alcohol consumption, and reactivation of chronic viral hepatitis (B, C, E) [2] are the most described precipitating factors. Other precipitating conditions are less common (approximately 8%), including acute alcoholic hepatitis, surgery, or TIPS insertion. Paracentesis without albumin substitution has also been described [4,9]. The main acute precipitating factors in ACLF, according to Gawande et al., are listed in Table 1 [10].

Evidence on ACLF caused by acute hepatic insults of a viral etiology is still being researched, further studies being essential for a concluding evaluation of etiology, outcome, organ failure, and mortality predictors. Hepatitis viruses are some of the main etiological factors for acute hepatic insults resulting in ACLF. Hepatotropic viral infections include hepatitis B virus reactivation, (HBV) superinfection with hepatitis A virus (HAV), hepatitis E virus (HEV), or more rarely, reactivation of HCV infection.

HBV reactivation has been recognized as a common factor involved in ACLF development [11,12,13]. Studies have suggested that 15–37% of patients with HBV infection develop acute exacerbations within four years [14], as well as episodes of ACLF; the mortality rate for these patients is between 30% and 70% [4,15]. In the Asia-Pacific region, hepatitis B appears to be the most common cause of ACLF. In China, 80% of ACLF cases are due to hepatitis B, and reactivation of HBV as an acute hepatic insult is found in more than 50% of cases [16].

HAV infection can occasionally cause liver failure in patients with or without preexisting chronic liver disease. Over time, a series of studies highlighted HAV as an acute insult in ACLF and provided evidence of increased short-term mortality [2,17]. 

Agrawal et al. reported the presence of ACLF and HAV as an acute insult that occurred in patients with underlying liver cirrhosis secondary to NAFLD (non-alcoholic fatty liver disease) [18]. Kahraman et al. also reported a human immunodeficiency virus (HIV)-positive case in a patient who developed ACLF due to an acute HAV infection with liver cirrhosis due to NAFLD [19]. Duseja et al. emphasized that HAV infection in patients with the pre-existing chronic liver disease could be responsible for worsening ACLF [20]. 

Other authors have investigated the role of HAV infection in patients with chronic hepatitis or liver cirrhosis secondary to HBV infection, suggesting that the association of HAV superinfection with HBV patients occasionally leads to ACLF, in HBV patients both with and without liver cirrhosis [21,22,23]. Some studies have associated HAV infection in HCV infection with increased mortality [24,25], although there have been several contrary opinions suggesting that HAV superinfection is associated with decreased HCV replication, which could lead to HCV clearance. [26,27,28].

Therefore, it is currently suggested that HAV infection, as an acute insult, precipitates the development of ACLF in patients with any chronic liver disease, especially liver cirrhosis, an important role being apparently attributed to host genetic factors determining different individual susceptibility [29,30].

Many studies have reported HEV as one of the leading causes of ACLF in Asia and Africa, where HEV is endemic. The mortality rate of HEV-related ACLF is nearly 35% [31]. HEV-related acute hepatitis in patients having underlying cirrhosis may complicate and worsen the primary disease and can result in ACLF. A majority of these patients may need early transplantation [32,33,34].

The evidence that, generally, HCV infection rarely causes ACLF has been supported by numerous previous studies [35,36,37,38,39,40], but a reactivation of HCV infection is recognized to be one of the precipitating factors in ACLF induction [9], considering the possibility that an acute HCV infection can result in acute fulminant liver failure [3,41,42,43].

Bacterial products, such as muramyl-dipeptides, bacterial DNA, lipopolysaccharides, and peptidoglycans, are translocated from a permeable intestine into the blood continuously or discontinuously; therefore, they could initiate initial organ dysfunction [44]. Bacterial translocation, the most important trigger of systemic inflammation, relies on the existence of ascites [45].

Gastrointestinal bleeding is also a precipitating factor in triggering ACLF. Patients with ACLF have a significantly increased bleeding history compared to patients without ACLF [46,47]. Rebleeding doubles the risk of ACLF, and these patients present a very high risk of death, so TIPS insertion could notably improve outcomes in critically decompensated cirrhotic patients [47,48,49]; therefore, TIPS has been recommended by the recent international guidelines and Baveno [50,51].

Host response is likely the main factor in determining ACLF severity and prognosis. The host immune reaction and the inflammatory cascade are of significance in this condition. The similarity between systemic inflammation determined by sepsis and ACLF motivates the idea that both conditions could have similar pathogenic mechanisms. Comparing patients with sepsis and patients with ACLF, Wasmuth et al. [52] formulated the hypothesis of “sepsis-like immune paralysis” based on extremely reduced TNF-α pro-duction and decreased HLA-DR monocyte expression in patients with both sepsis and ACLF, with this cellular immune damage contributing to high mortality.

Current studies have identified that ACLF can occur in patients with both compensated and decompensated cirrhosis, as well as in patients with previous CLD without cirrhosis. Table 2 presents the prevalence of ACLF in different CLD etiologies, according to data published in an exhaustive study conducted in 80,389 patients by Mahmud et al. [53]. 

**Figure 1:**
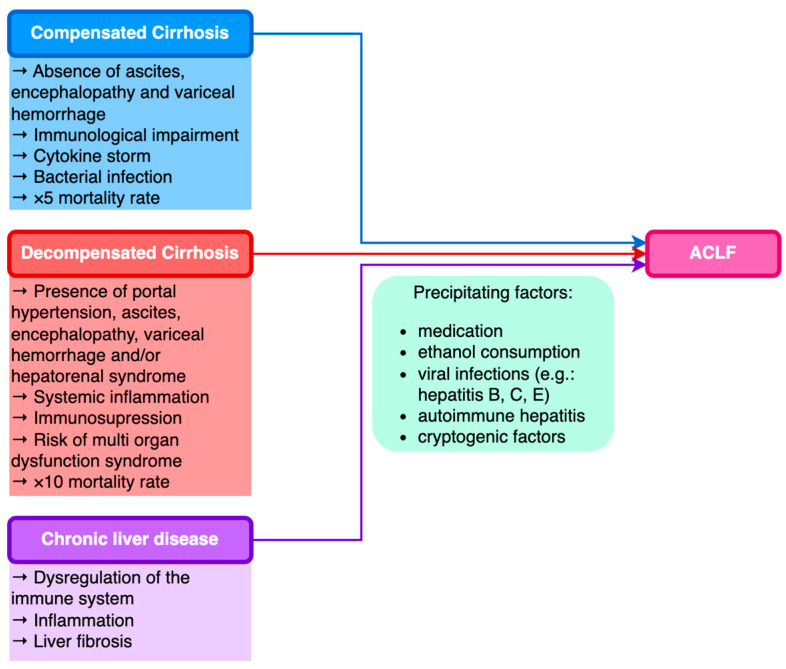
Classification of ACLF based on underlying chronic liver disease.

The World Gastroenterology Organization (WGO) formulated a classification of ACLF relying on the underlying liver disease: patients with underlying CLD and non-cirrhotic ACLF type A; patients with prior compensated cirrhosis and ACLF type B; and patients with prior decompensated cirrhosis and ACLF type C (Figure 1) [54,55].

Recent studies have revealed that ACLF represents 5% of all patients suffering from liver cirrhosis who are hospitalized, the average cost of hospitalization for these patients being three times higher than the cost for cirrhotic patients without complications and approximately five times higher than the cost for patients hospitalized for heart failure [56].

The global mortality rate according to EASL-CLIF is 30% to 50%. Mortality rates in the United States, according to NACSELD in decompensated patients, were 27%, 49%, 64%, and 77%, the percentage increasing directly with the number of organs affected (1, 2, 3, or 4 organ failures, respectively) [57,58].

## 3. Pathophysiological Hypotheses in ACLF

The pathophysiological hypothesis is determined by the idea that an acute trigger event causing hepatocyte injury in a patient with CLD determines a response characterized by an inflammatory cytokine reaction and, consequently, liver injury. Suppression of immune function and liver decompensation cause a concomitant increased risk of infections, associated with multi-organ failure and death [59].

### 3.1. Systemic Inflammation

Systemic inflammation, evidenced by leukocytosis and increased serum levels of cytokines and chemokines (including IL-6, IL-1, and IL-8), is commonly described in patients diagnosed with ACLF [59], these levels usually being absent in patients with liver cirrhosis without ACLF. Increased levels of IL-6 and IL-1 in patients diagnosed with acute decompensation were independently correlated with ACLF development within 28 days [44]. IL-1α, IL-1β, plasma renin, and copeptin are elevated in acute decompensation, as well as other cytokines (markers of inflammation), and they are significantly greatly associated with the onset of ACLF [60].

Nitric oxide, a strong vasoconstrictor mediator synthesized by the liver endothelium, has a significant role in the pathogenesis of ACLF, being able to neutralize the vasodilator stimuli released by cytokines [61].

The major systemic inflammatory response initiated in patients with ACLF can be influenced by exogenous factors, known as pathogen-associated molecular patterns (PAMPs), which originate from exogenous bacteria existing in the blood circulation, or by endogenous factors, known as damage-associated molecular patterns (DAMPs), which originate from damaged liver cells [62,63].

Among the causes of ACLF, bacterial infections are recognized as common causes; therefore, PAMPs originating from these bacteria could determine the inflammatory process [60,64]. Increased levels of circulating PAMPs are correlated with bacterial translocation from the gut and may promote the induction of ACLF without other diagnosed precipitating factors. PAMPs translocated from the intestine, as a result of an excessive increase in its permeability, will determine the alteration of the immune system function.

DAMPs are released into the blood circulation by damaged or necrotic cells or secondary to an extracellular matrix disruption, to make the immune system aware of the presence of tissue damage. DAMPs are identified by host receptors; their release determines sterile inflammation, (inflammation in the absence of evidence of infection) [65]. 

Liver injury is a recognized cause of DAMP release. In alcoholic hepatitis, hepatocyte apoptosis induced by alcohol is explained by multiple pathogenic mechanisms; abusive alcohol consumption reduces the mitochondrial maximal oxygen consumption rate, leading to increased susceptibility to hypoxia and acute liver injury [66,67]. 

### 3.2. Metabolic Dysfunction

Acute hepatic decompensation results in a hyper-metabolic status. Micronutrients (carbohydrates, amino acids, and fatty acids) are maintained in this status for immune cells with high metabolic need [68,69]. Nutrient deprivation, along with proinflammatory status, can induce mitochondrial dysfunction in vital organs, such as the kidney, heart, and liver, thus facilitating the development of ACLF [70,71].

Recent studies have highlighted the significant contribution of lipid metabolism in ACLF, represented by heterogeneous serum and tissue inflammatory mediators, synthesized by three enzyme families of cytochrome P450—cyclooxygenases, lipoxygenases, and epoxygenases. These enzymes generate a wide range of lipid mediators that promote inflammation, such as leukotriene E4. In contrast, the derived lipid mediator lipoxin A5 (LXA5), an anti-inflammatory mediator, is notably reduced in ACLF patients [69].

## 4. The Involvement of the Gut Microbiome in ACLF

The intestinal microbiome is significantly involved in the development of complications determined by liver cirrhosis [72].

Exacerbated intestinal permeability, endotoxin release, accumulation of intestinal bacteria, and bacterial toxin translocation predispose patients with liver cirrhosis to complications such as spontaneous bacterial peritonitis, hepatic encephalopathy, and ACLF. The intestinal microbiome is modified in patients with liver cirrhosis. There are significant data from quantitative metagenomic studies describing a multitude of different microbial genes in these patients compared to healthy individuals [73,74,75].

The gut microbiome is notably involved in metabolism, food digestion, vitamin synthesis, immune system function, and inflammation and cell proliferation [76,77]. Recent studies have shown alterations in the intestinal microbiome in liver afflictions [78].

Changes in the gut microbiome can play a role in the development of liver diseases through different mechanisms that include both environmental factors and genetics, as well as diet, bacterial structure, metabolism of bile acids, and factors increasing bacterial translocation through the increased permeable intestinal barrier into the portal venous system [79].

## 5. Biological Evaluation and Imaging in ACLF

Biological tests highlight elements of acute liver decompensation, such as increased bilirubin and aminotransferases, prolonged INR ≥ 1.5, anemia, thrombocytopenia, hypoglycemia, elevated serum ammonia, acute kidney injury (increased serum creatinine), and dyselectrolytemia (hypokalemia, hypophosphatemia, hyponatremia) [48,80,81,82].

Imaging evaluation methods are necessary to support the clinical diagnosis, to highlight an associated infection, and to evaluate organ failure. Brain, thoracic, abdominal, and pelvic imaging are thus recommended. Abdominal imaging confirms the signs of portal hypertension, possibly hepatocellular carcinoma, or thrombosis. Abdominal Doppler ultrasonography can be an alternative option in patients with concomitant renal lesions and vein thrombosis. Brain computed and magnetic resonance imaging is useful to exclude an organic cause of altered mental status, while chest imaging is useful in the diagnosis of pulmonary edema or pneumonia/bronchopneumonia.

## 6. ACLF Severity Classification

Being the most important cause of hospitalization in patients diagnosed with liver cirrhosis, the ACLF classification is useful for optimized management and for assessing prognosis. ACLF is divided into three grades according to its severity (Table 3) [4].

## 7. Management of ACLF

**Figure 2:**
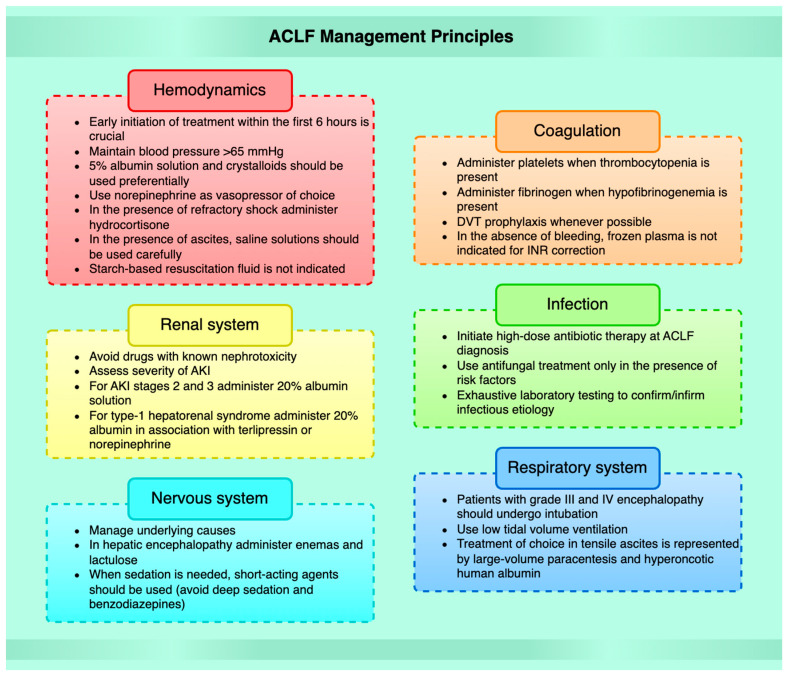
ACLF management principles. DVT, deep vein thrombosis. AKI, acute kidney injury.

The current management of ACLF involves primarily prevention of the trigger factors that lead to the acute development of liver decompensation, the support of vital functions, and the prevention and therapy of complications (Figure 2). It also consists of the assessment of the prognosis and the opportunity for liver transplantation [83].

Prevention of precipitating factors determining acute liver decompensation refers to all possible precipitating factors; thus, antibiotic treatment is initiated for any suspicion of an infectious process, as well as sustained therapy of chronic viral hepatitis (B or C) [84,85,86] and administration of albumin in spontaneous bacterial peritonitis to inhibit accelerated kidney damage characteristic of hepatorenal syndrome [87,88]. Albumin infusion is also acceptable and recognized in decompensated cirrhosis to correct hypovolemia and its associated complications [50]. Recent results of the ANSWER trial emphasized that achieving a serum albumin level of 4.0 g/dL correlates with a significant improvement in survival [89]. However, a randomized trial conducted by China et al., in patients hospitalized with liver cirrhosis, suggested that daily albumin infusion to preserve a serum albumin level ≥ 3 g/dL did not reveal any benefit in preventing the occurrence of infection, kidney injury, or death [90]. Conversely, there were more subjects in the albumin arm who presented with pulmonary edema and/or pulmonary infections [91].

In acute alcoholic hepatitis, administration is recommended of intravenous N-acetylcysteine, associated with corticotherapy (prednisolone) and pentoxifylline, with proven efficacy in reducing the development of type 1 hepatorenal syndrome.

Hemodynamic stability is ensured by administering intravenous infusion solutions and maintaining acid–base and electrolyte levels [92]. Vasopressor drugs are prescribed to preserve a mean blood pressure ≥ 75 mm Hg to preserve optimal cerebral and kidney perfusion [93].

The blood count is monitored to detect any possible bleeding because patients present with coagulopathy, thrombocytopenia, and platelet-altered functions. Administration of platelet mass and fresh frozen plasma is mainly indicated in selected patients with gastrointestinal bleeding or prior to an invasive procedure [81]. Proton pump inhibitors are administered for the prophylaxis of gastrointestinal bleeding.

In cases of hepatic encephalopathy, airway protection is necessary due to an increased risk of aspiration. 

It is necessary to preserve adequate nutrition with 1.0 to 1.5 g protein/kilogram/day [94,95,96]. Parenteral nutrition could be necessary in critical patients or in patients with severe hepatic encephalopathy with unprotected airways [97].

Hypoglycemia is monitored, maintaining serum glucose between 160 and 200 mg/dL.

### 7.1. Management of Viral Hepatitis-Related ACLF

Early implementation of antiviral therapy could improve the prognosis of HBV patients with ACLF. Antiviral therapy also increases the chances of stabilization until liver transplant time and improves transplant outcomes. Antivirals including lamivudine and entecavir (ETV) have shown short-term survival benefits in HBV-related patients with ACLF, despite the prevalence of drug resistance with lamivudine. Recently, a study demonstrated the role of tenofovir disoproxil fumarate (TDF) and ETV for the treatment of HBV-related ACLF and reported that the short-term efficacy and safety of TDF is higher than that of ETV [98]. Another study conducted by Li et al. revealed that HBV-related patients with ACLF treated with tenofovir alafenamide (TAF), TDF, and ETV have comparable 48-week survival without liver transplantation [99]. A study by Yang et al. suggested that initial combined use of antiviral drugs is efficient in reducing short-term mortality in HBV-related ACLF [100]. The APASL guidelines define the importance of early administration of antiviral therapy in HBV-related ACLF and suggest that those patients with chronic hepatitis B infection who need chemotherapy or immunosuppression procedures require rapid antiviral treatment to prevent consequences related to HBV reactivation [101]. 

ACLF rarely occurs in chronic hepatitis C without cirrhosis, unlike in non-cirrhotic HBV–ACLF [102,103]. HCV-related ACLF shows distinctive characteristics. Therefore, liver failure in HCV-related ACLF is very low (17.1%) compared to HBV-related ACLF (93.7%) [102]. Direct-acting antivirals (DAAs) have proved highly effective in patients with both chronic hepatitis C virus infection and liver cirrhosis, inducing a significant improvement in outcome and prognosis [104]. Sofosbuvir and other several DAAs, such as ledipasvir, daclatasvir, and simeprevir, or combined DAA therapy, including ombitasvir, paritaprevir, and ritonavir, contributed to high sustained virologic response rates [105,106,107,108]. Studies have indicated that DAA therapies ameliorate portal hypertension—a good predictor of liver-related events in cirrhosis, and in long-term follow-up, they improved metabolic liver function, regression of liver fibrosis, and amelioration of inflammation and macrophage activation, suggesting short- and long-term improvements in the outcomes, leading to an improved prognosis in patients with chronic hepatitis C and advanced liver disease [109,110]. 

Hepatitis E virus (HEV) infections can cause ALF in approximately 20–40% of cases in developing countries. In Asia, HEV infection is an important cause of ACLF. Nearly 21% of cases of ACLF in Asian countries are related to HEV infection, with high mortality within 28 days [31]. There is evidence for the use of pegylated interferon alpha and ribavirin in chronic HEV infection [111]. Ribavirin seems to be a possible treatment option in acute and chronic HEV, decreasing the severity of the disease in patients with both acute and chronic liver failure [112,113]. Further trials must be conducted to clarify the need and benefit of any antiviral treatment.

### 7.2. Management of Complications

Patients must be supported in the intensive care unit [114] to prevent the development and progression of multi-organ failure [115].

Kidney failure is secondary to hypovolemia and possibly to acute tubular necrosis or the occurrence of hepatorenal syndrome. Consequently, arterial hypotension is corrected by vasopressor therapy with norepinephrine or dopamine. Continuous renal replacement is preferred to hemodialysis [114].

In cases in which ACLF is determined by the presence of sepsis, broad-spectrum antibiotic therapy is instituted, and blood cultures, urine cultures, and sputum exams are performed [115].

Hypoglycemia secondary to the impairment of gluconeogenesis requires continuous administration of 10% or 20% glucose.

Hypophosphatemia, which occurs due to ATP consumption involved in hepatocyte necrosis, requires an adequate substitution. 

Alkalosis in ACLF occurs consequent to hyperventilation, while acidosis, having an increased severity, correlates with 95% mortality in the absence of liver transplantation.

Acute respiratory distress syndrome causes severe hypoxemia and occurs secondary to aspiration or pulmonary hemorrhage. Therefore, endotracheal intubation for airway protection is necessary in patients with grade ≥ 2 encephalopathy.

Encephalopathy is a crucial aspect of ACLF. Patients with increased arterial ammonia levels ≥ 200 µmol/L are at increased risk for intracranial hypertension. Cranial computed tomography should be performed in patients with severe encephalopathy to evaluate potential intracranial bleeding and the presence of cerebral edema [116,117]. The factors that determine the occurrence of cerebral edema are represented by hypoxia, arterial hypotension, and decreased cerebral perfusion pressure. Cerebral edema is the main cause of exitus in ACLF due to intracranial hypertension and ischemic brain lesions [116]. Endotracheal intubation and sedation are required in patients with grade 3 encephalopathy, as well as prompt administration of intravenous mannitol.

Coagulopathy is also a defining aspect of ACLF. Correction therapy of coagulopathy is recommended in cases of active gastrointestinal bleeding or prior to endoscopy or TIPS insertion. Platelets, frozen plasma, and cryoprecipitate transfusions may be recommended in distinct cases [58]. Administration of recombinant VII factor (off-label use) may cause thrombosis; intravenous vitamin K therapy may be indicated in cases of nutritional deficit or if prolonged cholestasis is associated.

### 7.3. Novel Therapeutic Strategies

#### 7.3.1. Granulocyte Colony-Stimulating Factor (GCSF)

In ACLF patients associated with severe sepsis, a therapeutic approach using a granulocyte colony-stimulating factor associated with darbepoetin seemed to improve one-year survival in patients diagnosed with decompensated cirrhosis [118,119]. A suggested G-CSF mechanism of action in ACLF therapy is represented by the mobilization of stem cells from the bone marrow and/or an increase in the number of liver progenitor cells. However, G-CSF potential therapy remains in the experimental stage, the results having to be confirmed by further studies [119].

#### 7.3.2. Mesenchymal Stem Cell Transplantation

Mesenchymal stem cells (MSCs) are pluripotent cells able to regenerate and differentiate into different cell types, including hepatocytes [120]. A meta-analysis evaluating the clinical efficacy of MSC treatment in the management of ACLF found a short-term correction of biological liver tests and an improvement in the MELD score [121]. Thus, it is expected that MSC treatment in ACLF would have potentially beneficial effects. Stem cell treatment could represent a future therapeutic approach in patients with ACLF, but evidence to sustain MSC therapy in clinical practice requires further studies. 

#### 7.3.3. Inhibition of Toll-like Receptor 4 (TLR-4); TAK-242

Immune cell activation mediated by TLR makes a crucial contribution to the innate immune response, thus contributing to the pathogenesis of ACLF [122]. By recognizing PAMPs, and DAMPs, TLRs promote the innate immune cells, including macrophages and neutrophils, thereby stimulating inflammation [123]. TAK-242 inhibits TLR-4 activation [124], thus implicitly blocking DAMPs in initiating the release of pro-inflammatory cytokines. Experimental studies have encouraged these hypotheses.

#### 7.3.4. Oxysterol Sulfates: 25-Hydroxycholesterol 3-Sulfate (25HC3S)

Oxysterol sulfates represent new potential anti-inflammatory drugs with important roles in both lipid metabolism and inflammatory responses due to epigenetic changes. Their role in the management of ACLF needs to be proved in large future studies [125]. 

#### 7.3.5. Recombinant Alkaline Phosphatase

A therapeutic perspective in ACLF consists of blocking TLR4 by neutralizing PAMPs and lipopolysaccharide endotoxin (LPS), an endotoxin from Gram-negative bacteria cellular membrane, which can be neutralized by dephosphorylation mediated by recombinant alkaline phosphatase [126]. The motivation of these therapies is based on studies that have shown an increase in the serum level of LPS in patients with decompensated cirrhosis and ACLF, which are associated with a poor prognosis and high mortality [127]. 

#### 7.3.6. Mitofusin-2

Mitochondrial fusion protein 2 is involved in the mechanisms of apoptosis and autophagy [128]. Since hepatocytic necrosis is probably the key factor in ACLF, mitofusin-2, by reducing apoptosis, could become a potential element in future combined therapies in ACLF patients [129]. 

#### 7.3.7. Statins

Statins represent a class of drugs used in the treatment of hyperlipidemia, but they have anti-inflammatory, antioxidant, and antiproliferative effects [130]. Statins have a had proven effect in multiple studies to increase the release of nitric oxide in the liver, decreasing vascular resistance in patients with liver cirrhosis, portal hypertension, and ascites. Experimental studies conducted by Trebicka and Marrone demonstrated hepatoprotective and anti-inflammatory properties of statins [131,132], and Pollo et al. showed that statins decreased portal pressure in patients with liver cirrhosis, with favorable effects on survival in patients presenting with variceal bleeding [133].

#### 7.3.8. N-Acetylcysteine

N-acetylcysteine (NAC) represents the main treatment in paracetamol-induced ALF [134]. NAC has an anti-oxidant effect and favors the elimination of free radicals, increasing mitochondrial function, inhibiting inflammation, improving liver function, and promoting hepatocyte regeneration [135]. Wang et al. studied the efficacy of NAC treatment in patients with viral B hepatitis and ACLF in a retrospective study conducted in 90 patients, which showed that NAC improved biological liver tests, coagulation function, and intrahepatic cholestasis [136]. 

New strategies of treatment are illustrated in ongoing trials: NCT04229901, phase II, evaluates the use of Hepa Stem in inhibiting hepatic stellate cells activation, reducing collagen secretion and downregulation of pro-inflammatory environment; NCT04822922, phase II, evaluates umbilical cord-derived MSCs in improving liver fibrosis and regeneration; NCT03780673, phase IIII, evaluates the combination of simvastatin plus rifaximin in reducing HSC activation and proliferation, increasing liver sinusoidal function and decreasing inflammation, thus preventing hepatic encephalopathy; and NCT01698723, phase II, evaluates ribavirin treatment in hepatitis E virus infection. The management of excessive inflammation is also supported for better outcomes of ACLF since systemic inflammation is a main factor in determining ACLF prognosis [137]. These new strategies require further research to prove their efficacy in ACLF management.

A synthesis of the main key strategies in the management of ACLF was provided by Bajaj et al. in 2022 [5]. 

### 7.4. Liver Support and Transplantation

Although a liver transplant (LT) is the only absolute treatment for ACLF, there are no clear definitions in the selection criteria [138,139]. Bajaj et al. suggested in their guidelines that EASL-CLIF score may be useful for prioritizing patients eligible for LT, while the NASLED scoring system is better suited for exclusion of patients from this procedure [5].

Certain concerns, such as alcohol recidivism or a reduction in physiological reserve, still prevent LT from being universally accepted as a treatment of ACLF. However, data have provided overwhelming evidence for incorporating LT into the management of these patients. The CANONIC study revealed that LT led to an increase in survival rate from 20% to 80%. Other data suggest that one-year survival after LT for ACLF exceeds 70% [140].

Studies assessing prognosis after LT in patients with liver failure have proven favorable outcomes after LT but should be carefully interpreted due to the strict selection for transplant only in patients who are most likely to achieve good prognosis [141,142].

Extracorporeal liver support devices were tested in clinical trials in patients with liver failure and could represent a potentially beneficial treatment. Extra-corporeal liver support devices improve liver function [143]. However, recent multicenter studies have not shown increased survival rates [144,145].

In patients with hepatitis B and ACLF, plasma exchange was followed by an improvement in short-term survival [146].

## 8. Prognosis of ACLF

ACLF has an increased mortality rate of 50% to 90%. Discrepancies and non-uniformity in the terminology of ACLF and therefore the different features of the studied patients have limited the possibilities of the identification of certain indicators of severity and prognosis [147]. 

The impact of ACLF on cirrhotic and non-cirrhotic patients has varied among different studies. A study conducted by Thanapirom et al. in 2021 in 1621 enrolled patients, which evaluated the impact of compensated cirrhosis on survival in patients with ACLF, showed that short-term mortality rates of ACLF in patients without cirrhosis were significantly increased compared to patients with alcoholic liver cirrhosis [148]. Shalimar et al. found that alcohol liver injury was an independent risk factor related to increased mortality [149].

The presence of cirrhosis was an independent factor in mortality, and MELD scores, organ failure, variceal bleeding, and hepatic encephalopathy were associated with unfavorable prognosis [150,151].

Distinct effects of each condition might be explained by the heterogeneity of study protocols and ACLF definition among different studies [55].

Several prognostic models already widely described for liver cirrhosis patients have been applied to assess this syndrome’s prognosis. For this reason, the prognostic scores are classified into two categories: the first assesses the severity of liver dysfunction (Child-Pugh, MELD), and the second assess global prognostic scores (Evaluation of Acute and Chronic Physiology of Health (APACHE II; SOFA). Recent studies have highlighted that global prognostic scores are superior for predicting prognosis in ACLF patients [152]. APACHE-II seems to be the best predictive scoring system, according to the hypothesis that in ACLF, once liver failure is diagnosed, the prognosis is determined by the severity of other organ dysfunction and not by the severity of liver failure [153]. According to recent studies, the MELD score was described to be comparable to SOFA and APACHE II [154]. 

Recently, based on evidence from the CANONIC study, a specific score for ACLF prognosis, called the “CLIF-CONSORTIUM score for ACLF” (CLIF-C ACLF score), was developed. This score is the result of combining the “CLIF-Consortium Organ Failure (CLIF-COF)” score (used for the diagnosis of ACLF) and two other independent predictors of mortality (age and leukocyte count) [54,147]. The CLIF-C ACLF score has been also considered a distinct factor of severity [83].

## 9. Prevention of ACLF

Prevention of ACLF consists of the identification of predisposing conditions, together with clinical examination and biological and imaging investigations.

Prevention of ACLF is particularly difficult due to the severity of the syndrome and its clinical different features and complications. Cohort studies (CANONIC, NACSELD) and group consensuses (APASL, WGO) have helped in proving the diagnosis. Current management options for ACLF represent actually prevalent tertiary prevention and, in some cases, secondary prevention. In the days immediately following ACLF diagnosis, the clinical and biological status will be assessed and evaluated, and the opportunity for specific intensive therapy support will be evaluated, including evaluation for liver transplantation (tertiary prevention). In cases with single organ involvement, the target is to prevent the subsequent occurrence of another organ failure by addressing aggressive intensive medical therapy (e.g., antibiotics prescribed to prevent complications, such as hepatorenal syndrome). 

Prevention of other organ failure than the liver is an example of secondary prevention. It is the main target in the management of ACLF. To prevent the appearance of the syndrome (primary prevention), APASL observed a “golden window”, a short period of approximately seven days before the onset of sepsis and the development of insufficiency of extrahepatic organs (kidneys, brain, circulatory system, or pulmonary damage), in patients with ACLF. Early therapy during this time is responsible for preventing the onset of organ failure and possibly the development of ACLF (‘primary prophylaxis’). Currently, early diagnosis, history, laboratory examinations, and imaging methods are the only methods capable of detecting this “golden window” [62].

Prevention of ACLF should consider therapeutic strategies that target the main pathophysiological mechanisms participating in the initiation of ACLF. Recent studies have revealed that pathogenic mechanisms are mainly represented by the characteristic damage to the gut–liver axis determining bacterial translocation and systemic inflammation [62]. Therefore, therapeutic options in bacterial translocation and those that modulate the inflammatory response (antibiotics, albumin, statins) represent the main therapeutic principles that should be proposed as first-line defense treatments for the development of ACLF.

## 10. Conclusions

ACLF is a distinct condition from simple cirrhosis decompensation, with different clinical and physio-pathological features, and ACLF is a devastating, life-threatening syndrome. Systemic inflammation, associated with metabolic dysfunction, is the key physiopathological mechanism that contributes to the development of this severe condition. Prevention of major trigger factors, such as infections or inappropriate alcohol consumption, is essential to improve the prognosis of organ failure. Care management strategies in the intensive care unit and the stratification of cases that could benefit from liver transplants are essential, depending on the individualized prognosis of each individual case. Liver transplant remains the last resort in the management of patients suffering from liver failure and the only curative method known, but high costs and low availability severely limit patients’ access to this therapy. Early recognition of this serious condition associated with intensive, supportive treatment could contribute to lowering the number of liver transplant recipients, thus reducing the exhaustive expenses associated with this procedure.

## Figures and Tables

**Table 1:** Acute precipitating factors in ACLF (adaptation after Gawande et al. [10]).

Precipitating Factor	Prevalence
Alcohol	48.08%
Sepsis	16.35%
Gastrointestinal bleeding	19.13%
HBV	8.2%
HEV	7.21%
Tuberculostatic treatment	6.25%
Autoimmune hepatitis	0.96%
Unknown	3.85%

**Table 2:** Prevalence of ACLF based on etiology of CLD (according to data published by Mahmud et al. [53]).

Etiology	Total Patients	ACLF
Hepatitis C	13,959	809 (5.79%)
Hepatitis B	1967	145 (7.37%)
Alcohol	23,484	1798 (7.65%)
Hepatitis C + Alcohol	22,343	1791 (8.01%)
NAFLD	15,893	929 (5.84%)
Other	2737	181 (6.61%)

NAFLD, non-alcoholic fatty liver disease.

**Table 3:** Grading of ACLF severity (according to Moreau et al. [4]).

ACLF Grade	Characteristics	Mortality Rate (28 Days)
1	→kidney failure OR →liver failure, coagulation disorder, or pulmonary impairment + serum creatinine level 1.5–1.9 mg/dL OR →hepatic encephalopathy of grade 1 or 2 ± serum creatinine level 1.5–1.9 mg/dL	22.1%
2	→presence of at least two combined organ failures	32.0%
3	→three or more organ failures are implied	76.7%

## Data Availability

The dataset presented in this study is available from the corresponding author upon reasonable request.

## References

[1] O’Leary J.G., Reddy K.R., Garcia-Tsao G., Biggins S.W., Wong F., Fallon M.B., Subramanian R.M., Kamath P.S., Thuluvath P., Vargas H.E. (2018). NACSELD Acute-on-Chronic Liver Failure (NACSELD-ACLF) Score Predicts 30-Day Survival in Hospitalized Patients with Cirrhosis. Hepatology.

[2] Sarin S.K., Choudhury A., Sharma M.K., Maiwall R., Al Mahtab M., Rahman S., Saigal S., Saraf N., Soin A.S., Devarbhavi H. (2019). Acute-on-Chronic Liver Failure: Consensus Recommendations of the Asian Pacific Association for the Study of the Liver (APASL): An Update. Hepatol. Int..

[3] Sarin S.K., Kedarisetty C.K., Abbas Z., Amarapurkar D., Bihari C., Chan A.C., Chawla Y.K., Dokmeci A.K., Garg H., Ghazinyan H. (2014). Acute-on-Chronic Liver Failure: Consensus Recommendations of the Asian Pacific Association for the Study of the Liver (APASL) 2014. Hepatol. Int..

[4] Moreau R., Jalan R., Gines P., Pavesi M., Angeli P., Cordoba J., Durand F., Gustot T., Saliba F., Domenicali M. (2013). Acute-on-Chronic Liver Failure Is a Distinct Syndrome That Develops in Patients with Acute Decompensation of Cirrhosis. Gastroenterology.

[5] Bajaj J.S., O’Leary J.G., Lai J.C., Wong F., Long M.D., Wong R.J., Kamath P.S. (2022). Acute-on-Chronic Liver Failure Clinical Guidelines. Off. J. Am. Coll. Gastroenterol. ACG.

[6] Engelmann C., Thomsen K.L., Zakeri N., Sheikh M., Agarwal B., Jalan R., Mookerjee R.P. (2018). Validation of CLIF-C ACLF Score to Define a Threshold for Futility of Intensive Care Support for Patients with Acute-on-Chronic Liver Failure. Crit. Care.

[7] Engelmann C., Berg T. (2023). Clinical Practice Guidelines for Acute-on-Chronic Liver Failure: Are We Ready for Reaching Global Consensus?. Hepatobiliary Surg. Nutr..

[8] Trebicka J., Reiberger T., Laleman W. (2018). Gut-Liver Axis Links Portal Hypertension to Acute-on-Chronic Liver Failure. Visc. Med..

[9] Kim T.Y., Kim D.J. (2013). Acute-on-Chronic Liver Failure. Clin. Mol. Hepatol..

[10] Gawande A., Gupta G.K., Gupta A., Wanjari S.J., Goel V., Rathore V., Bhardwaj H., Nijhawan S. (2019). Acute-on-Chronic Liver Failure: Etiology of Chronic and Acute Precipitating Factors and Their Effect on Mortality. J. Clin. Exp. Hepatol..

[11] Li H., Chen L.-Y., Zhang N., Li S.-T., Zeng B., Pavesi M., Amorós À., Mookerjee R.P., Xia Q., Xue F. (2016). Characteristics, Diagnosis and Prognosis of Acute-on-Chronic Liver Failure in Cirrhosis Associated to Hepatitis B. Sci. Rep..

[12] Yin S., Wang S.J., Gu W.Y., Zhang Y., Chen L.Y., Li H. (2017). Risk of Different Precipitating Events for Progressing to Acute-on-Chronic Liver Failure in HBV-Related Cirrhotic Patients. J. Dig. Dis..

[13] Shi Y., Yang Y., Hu Y., Wu W., Yang Q., Zheng M., Zhang S., Xu Z., Wu Y., Yan H. (2015). Acute-on-chronic Liver Failure Precipitated by Hepatic Injury Is Distinct from That Precipitated by Extrahepatic Insults. Hepatology.

[14] Kumar M., Chauhan R., Gupta N., Hissar S., Sakhuja P., Sarin S.K. (2009). Spontaneous Increases in Alanine Aminotransferase Levels in Asymptomatic Chronic Hepatitis B Virus-Infected Patients. Gastroenterology.

[15] Sarin S.K., Kumar A., Almeida J.A., Chawla Y.K., Fan S.T., Garg H., de Silva H.J., Hamid S.S., Jalan R., Komolmit P. (2009). Acute-on-Chronic Liver Failure: Consensus Recommendations of the Asian Pacific Association for the Study of the Liver (APASL). Hepatol. Int..

[16] Wan Z., Wu Y., Yi J., You S., Liu H., Sun Z., Zhu B., Zang H., Li C., Liu F. (2015). Combining Serum Cystatin C with Total Bilirubin Improves Short-Term Mortality Prediction in Patients with HBV-Related Acute-On-Chronic Liver Failure. PLoS ONE.

[17] Radha Krishna Y., Saraswat V.A., Das K., Himanshu G., Yachha S.K., Aggarwal R., Choudhuri G. (2009). Clinical Features and Predictors of Outcome in Acute Hepatitis A and Hepatitis E Virus Hepatitis on Cirrhosis. Liver Int..

[18] Agrawal S., Rana B.S., Mitra S., Duseja A., Das A., Dhiman R.K., Chawla Y. (2018). A Case of Acute-on-Chronic Liver Failure (ACLF) Due to An Uncommon Acute And Chronic Event. J. Clin. Exp. Hepatol..

[19] Kahraman A., Miller M., Gieseler R.K., Gerken G., Scolaro M.J., Canbay A. (2006). Non-Alcoholic Fatty Liver Disease in HIV-Positive Patients Predisposes for Acute-on-Chronic Liver Failure: Two Cases. Eur. J. Gastroenterol. Hepatol..

[20] Duseja A., Chawla Y.K., Dhiman R.K., Kumar A., Choudhary N., Taneja S. (2010). Non-Hepatic Insults Are Common Acute Precipitants in Patients with Acute on Chronic Liver Failure (ACLF). Dig. Dis. Sci..

[21] Zhang X., Ke W., Xie J., Zhao Z., Xie D., Gao Z. (2010). Comparison of Effects of Hepatitis E or A Viral Superinfection in Patients with Chronic Hepatitis B. Hepatol. Int..

[22] Fu J., Guo D., Gao D., Huang W., Li Z., Jia B. (2016). Clinical Analysis of Patients Suffering from Chronic Hepatitis B Superinfected with Other Hepadnaviruses. J. Med. Virol..

[23] Beisel C., Addo M.M., Schulze zur Wiesch J. (2020). Seroconversion of HBsAG Coincides with Hepatitis A Super-Infection: A Case Report. World J. Clin. Cases.

[24] Spada E., Genovese D., Tosti M.E., Mariano A., Cuccuini M., Proietti L., Giuli C.D., Lavagna A., Crapa G.E., Morace G. (2005). An Outbreak of Hepatitis A Virus Infection with a High Case-Fatality Rate among Injecting Drug Users. J. Hepatol..

[25] Lefilliatre P., Villeneuve J.-P. (2000). Fulminant Hepatitis A in Patients with Chronic Liver Disease. Can. J. Public Health.

[26] Sagnelli E., Coppola N., Pisaturo M., Pisapia R., Onofrio M., Sagnelli C., Catuogno A., Scolastico C., Piccinino F., Filippini P. (2006). Clinical and Virological Improvement of Hepatitis B Virus—Related or Hepatitis C Virus—Related Chronic Hepatitis with Concomitant Hepatitis A Virus Infection. Clin. Infect. Dis..

[27] Deterding K., Tegtmeyer B., Cornberg M., Hadem J., Potthoff A., Böker K.H.W., Tillmann H.L., Manns M.P., Wedemeyer H. (2006). Hepatitis A Virus Infection Suppresses Hepatitis C Virus Replication and May Lead to Clearance of HCV. J. Hepatol..

[28] Cacopardo B., Nunnari G., Nigro L. (2009). Clearance of HCV RNA Following Acute Hepatitis A Superinfection. Dig. Liver Dis..

[29] Kashyap P., Deka M., Medhi S., DUTTA S., Kashyap K., Kumari N. (2018). Association of Toll-like Receptor 4 with Hepatitis A Virus Infection in Assam. Acta Virol..

[30] Rubicz R., Yolken R., Drigalenko E., Carless M.A., Dyer T.D., Kent Jr J., Curran J.E., Johnson M.P., Cole S.A., Fowler S.P. (2015). Genome-Wide Genetic Investigation of Serological Measures of Common Infections. Eur. J. Hum. Genet..

[31] Kumar A., Saraswat V.A. (2013). Hepatitis E and Acute-on-Chronic Liver Failure. J. Clin. Exp. Hepatol..

[32] Choi J.W., Son H.J., Lee S.S., Jeon H., Cho J.-K., Kim H.J., Cha R.R., Lee J.M., Kim H.J., Jung W.T. (2022). Acute Hepatitis E Virus Superinfection Increases Mortality in Patients with Cirrhosis. BMC Infect. Dis..

[33] Kmush B., Wierzba T., Krain L., Nelson K., Labrique A.B. (2013). Epidemiology of Hepatitis E in Low- and Middle-Income Countries of Asia and Africa. Semin. Liver Dis..

[34] Artru F., Louvet A., Ruiz I., Levesque E., Labreuche J., Ursic-Bedoya J., Lassailly G., Dharancy S., Boleslawski E., Lebuffe G. (2017). Liver Transplantation in the Most Severely Ill Cirrhotic Patients: A Multicenter Study in Acute-on-Chronic Liver Failure Grade 3. J. Hepatol..

[35] Kanda T., Matsumoto N., Ishii T., Arima S., Shibuya S., Honda M., Sasaki-Tanaka R., Masuzaki R., Kanezawa S., Nishizawa T. (2023). Chronic Hepatitis C: Acute Exacerbation and Alanine Aminotransferase Flare. Viruses.

[36] Kanda T., Yokosuka O., Imazeki F., Saisho H. (2004). Acute Hepatitis C Virus Infection, 1986-2001: A Rare Cause of Fulminant Hepatitis in Chiba, Japan. Hepatogastroenterology.

[37] Liang T.J., Jeffers L., Reddy R.K., Silva M.O., Cheinquer H., Findor A., De Medina M., Yarbough P.O., Reyes G.R., Schiff E.R. (1993). Fulminant or Subfulminant Non-A, Non-B Viral Hepatitis: The Role of Hepatitis C and E Viruses. Gastroenterology.

[38] Jain A., Kar P., Madan K., Das U.P., Budhiraja S., Gopalkrishna V., Sharma J.K., Das B.C. (1999). Hepatitis C Virus Infection in Sporadic Fulminant Viral Hepatitis in North India: Cause or Co-Factor?. Eur. J. Gastroenterol. Hepatol..

[39] Maheshwari A., Ray S., Thuluvath P.J. (2008). Acute Hepatitis C. Lancet.

[40] Rao A., Rule J.A., Cerro-Chiang G., Stravitz R.T., McGuire B.M., Lee G., Fontana R.J., Lee W.M. (2023). Role of Hepatitis C Infection in Acute Liver Injury/Acute Liver Failure in North America. Dig. Dis. Sci..

[41] Younis B.B., Arshad R., Khurhsid S., Masood J., Nazir F., Tahira M. (2015). Fulminant Hepatic Failure (FHF) Due to Acute Hepatitis C. Pak. J. Med. Sci..

[42] Sagnelli E., Pisaturo M., Stanzione M., Messina V., Alessio L., Sagnelli C., Starace M., Pasquale G., Coppola N. (2013). Clinical Presentation, Outcome, and Response to Therapy among Patients with Acute Exacerbation of Chronic Hepatitis C. Clin. Gastroenterol. Hepatol..

[43] Thiel A.M., Rissland J., Lammert F., Casper M. (2018). Acute liver failure as a rare case of a frequent disease. Z. Gastroenterol..

[44] Trebicka J., Amoros A., Pitarch C., Titos E., Alcaraz-Quiles J., Schierwagen R., Deulofeu C., Fernandez-Gomez J., Piano S., Caraceni P. (2019). Addressing Profiles of Systemic Inflammation Across the Different Clinical Phenotypes of Acutely Decompensated Cirrhosis. Front. Immunol..

[45] Wiest R., Lawson M., Geuking M. (2014). Pathological Bacterial Translocation in Liver Cirrhosis. J. Hepatol..

[46] Trebicka J., Gu W., Ibáñez-Samaniego L., Hernández-Gea V., Pitarch C., Garcia E., Procopet B., Giráldez Á., Amitrano L., Villanueva C. (2020). Rebleeding and Mortality Risk Are Increased by ACLF but Reduced by Pre-Emptive TIPS. J. Hepatol..

[47] Costa D., Simbrunner B., Jachs M., Hartl L., Bauer D., Paternostro R., Schwabl P., Scheiner B., Stättermayer A.F., Pinter M. (2021). Systemic Inflammation Increases across Distinct Stages of Advanced Chronic Liver Disease and Correlates with Decompensation and Mortality. J. Hepatol..

[48] Hernández-Gea V., Procopet B., Giráldez Á., Amitrano L., Villanueva C., Thabut D., Ibañez-Samaniego L., Silva-Junior G., Martinez J., Genescà J. (2019). Preemptive-TIPS Improves Outcome in High-Risk Variceal Bleeding: An Observational Study. Hepatology.

[49] Kumar R., Kerbert A.J.C., Sheikh M.F., Roth N., Calvao J.A.F., Mesquita M.D., Barreira A.I., Gurm H.S., Ramsahye K., Mookerjee R.P. (2021). Determinants of Mortality in Patients with Cirrhosis and Uncontrolled Variceal Bleeding. J. Hepatol..

[50] European Association for the Study of the Liver (2018). Electronic address: Easloffice@easloffice.eu; European Association for the Study of the Liver EASL Clinical Practice Guidelines for the Management of Patients with Decompensated Cirrhosis. J. Hepatol..

[51] Gerbes A.L., Labenz J., Appenrodt B., Dollinger M., Gundling F., Gülberg V., Holstege A., Lynen-Jansen P., Steib C.J., Trebicka J. (2019). Aktualisierte S2k-Leitlinie der Deutschen Gesellschaft für Gastroenterologie, Verdauungs- und Stoffwechselkrankheiten (DGVS) „Komplikationen der Leberzirrhose“: AWMF-Nr.: 021-017. Z. Gastroenterol..

[52] Wasmuth H.E., Kunz D., Yagmur E., Timmer-Stranghöner A., Vidacek D., Siewert E., Bach J., Geier A., Purucker E.A., Gressner A.M. (2005). Patients with Acute on Chronic Liver Failure Display ‘Sepsis-like’ Immune Paralysis. J. Hepatol..

[53] Mahmud N., Kaplan D.E., Taddei T.H., Goldberg D.S. (2019). Incidence and Mortality of Acute-on-Chronic Liver Failure Using Two Definitions in Patients with Compensated Cirrhosis. Hepatology.

[54] Jalan R., Yurdaydin C., Bajaj J.S., Acharya S.K., Arroyo V., Lin H.-C., Gines P., Kim W.R., Kamath P.S. (2014). Toward an Improved Definition of Acute-on-Chronic Liver Failure. Gastroenterology.

[55] Wlodzimirow K.A., Eslami S., Abu-Hanna A., Nieuwoudt M., Chamuleau R.A.F.M. (2013). A Systematic Review on Prognostic Indicators of Acute on Chronic Liver Failure and Their Predictive Value for Mortality. Liver Int..

[56] Singer C.E., Vasile C.M., Popescu M., Popescu A.I.S., Marginean I.C., Iacob G.A., Popescu M.D., Marginean C.M. (2023). Role of Iron Deficiency in Heart Failure—Clinical and Treatment Approach: An Overview. Diagnostics.

[57] Avolio A.W., Gaspari R., Teofili L., Bianco G., Spinazzola G., Soave P.M., Paiano G., Francesconi A.G., Arcangeli A., Nicolotti N. (2019). Postoperative Respiratory Failure in Liver Transplantation: Risk Factors and Effect on Prognosis. PLoS ONE.

[58] Mattos D.Â.Z., Mattos D.A.A. (2019). Letter to the Editor: Acute-on-Chronic Liver Failure: Conceptual Divergences. Hepatology.

[59] Trebicka J., Fernandez J., Papp M., Caraceni P., Laleman W., Gambino C., Giovo I., Uschner F.E., Jansen C., Jimenez C. (2021). PREDICT Identifies Precipitating Events Associated with the Clinical Course of Acutely Decompensated Cirrhosis. J. Hepatol..

[60] Clària J., Stauber R.E., Coenraad M.J., Moreau R., Jalan R., Pavesi M., Amorós À., Titos E., Alcaraz-Quiles J., Oettl K. (2016). Systemic Inflammation in Decompensated Cirrhosis: Characterization and Role in Acute-on-Chronic Liver Failure. Hepatology.

[61] Thabut D., Tazi K.A., Bonnefont-Rousselot D., Aller M., Farges O., Guimont M.C., Tellier Z., Guichard C., Ogier-Denis E., Poynard T. (2007). High-Density Lipoprotein Administration Attenuates Liver Proinflammatory Response, Restores Liver Endothelial Nitric Oxide Synthase Activity, and Lowers Portal Pressure in Cirrhotic Rats. Hepatology.

[62] Hernaez R., Solà E., Moreau R., Ginès P. (2017). Acute-on-Chronic Liver Failure: An Update. Gut.

[63] Clària J., Arroyo V., Moreau R. (2016). The Acute-on-Chronic Liver Failure Syndrome, or When the Innate Immune System Goes Astray. J. Immunol..

[64] Arroyo V., Moreau R., Kamath P.S., Jalan R., Ginès P., Nevens F., Fernández J., To U., García-Tsao G., Schnabl B. (2016). Acute-on-Chronic Liver Failure in Cirrhosis. Nat. Rev. Dis. Prim..

[65] Kubes P., Mehal W.Z. (2012). Sterile Inflammation in the Liver. Gastroenterology.

[66] Medzhitov R. (2008). Origin and Physiological Roles of Inflammation. Nature.

[67] Petrasek J., Iracheta-Vellve A., Csak T., Satishchandran A., Kodys K., Kurt-Jones E.A., Fitzgerald K.A., Szabo G. (2013). STING-IRF3 Pathway Links Endoplasmic Reticulum Stress with Hepatocyte Apoptosis in Early Alcoholic Liver Disease. Proc. Natl. Acad. Sci. USA.

[68] Ganeshan K., Nikkanen J., Man K., Leong Y.A., Sogawa Y., Maschek J.A., Van Ry T., Chagwedera D.N., Cox J.E., Chawla A. (2019). Energetic Trade-Offs and Hypometabolic States Promote Disease Tolerance. Cell.

[69] López-Vicario C., Checa A., Urdangarin A., Aguilar F., Alcaraz-Quiles J., Caraceni P., Amorós A., Pavesi M., Gómez-Cabrero D., Trebicka J. (2020). Targeted Lipidomics Reveals Extensive Changes in Circulating Lipid Mediators in Patients with Acutely Decompensated Cirrhosis☆. J. Hepatol..

[70] Van Wyngene L., Vandewalle J., Libert C. (2018). Reprogramming of Basic Metabolic Pathways in Microbial Sepsis: Therapeutic Targets at Last?. EMBO Mol. Med..

[71] Moreau R., Clària J., Aguilar F., Fenaille F., Lozano J.J., Junot C., Colsch B., Caraceni P., Trebicka J., Pavesi M. (2020). Blood Metabolomics Uncovers Inflammation-Associated Mitochondrial Dysfunction as a Potential Mechanism Underlying ACLF. J. Hepatol..

[72] Gómez-Hurtado I., Such J., Sanz Y., Francés R. (2014). Gut Microbiota-Related Complications in Cirrhosis. World J. Gastroenterol..

[73] Qin N., Yang F., Li A., Prifti E., Chen Y., Shao L., Guo J., Le Chatelier E., Yao J., Wu L. (2014). Alterations of the Human Gut Microbiome in Liver Cirrhosis. Nature.

[74] Gosalbes M.J., Abellan J.J., Durbán A., Pérez-Cobas A.E., Latorre A., Moya A. (2012). Metagenomics of Human Microbiome: Beyond 16s RDNA. Clin. Microbiol. Infect..

[75] Arumugam M., Raes J., Pelletier E., Le Paslier D., Yamada T., Mende D.R., Fernandes G.R., Tap J., Bruls T., Batto J.-M. (2011). Enterotypes of the Human Gut Microbiome. Nature.

[76] Doré J., Simrén M., Buttle L., Guarner F. (2013). Hot Topics in Gut Microbiota. United Eur. Gastroenterol. J..

[77] Acharya C., Bajaj J.S. (2019). Altered Microbiome in Patients With Cirrhosis and Complications. Clin. Gastroenterol. Hepatol..

[78] Schwenger K.J., Clermont-Dejean N., Allard J.P. (2019). The Role of the Gut Microbiome in Chronic Liver Disease: The Clinical Evidence Revised. JHEP Rep..

[79] Duseja A., Chawla Y.K. (2014). Obesity and NAFLD: The Role of Bacteria and Microbiota. Clin. Liver Dis..

[80] Artzner T., Michard B., Weiss E., Barbier L., Noorah Z., Merle J.-C., Paugam-Burtz C., Francoz C., Durand F., Soubrane O. (2020). Liver Transplantation for Critically Ill Cirrhotic Patients: Stratifying Utility Based on Pretransplant Factors. Am. J. Transpl..

[81] Inherited Metabolic Liver Disease Collaboration Group, Chinese Society of Hepatology, Chinese Medical Association (2022). [Guidelines for the diagnosis and treatment of hepatolenticular degeneration (2022 edition)]. Zhonghua Gan Zang Bing Za Zhi.

[82] Steve R.J., Gnanadurai F.J., Anantharam R., Jeyaseelan V., Zachariah U.G., Goel A., Chundamannil E.E., Abraham P. (2018). Expanded Diagnostic Approach to Hepatitis E Virus Detection in Patients with Acute-on-Chronic Liver Failure: A Pilot Study. Indian J. Med. Microbiol..

[83] Arroyo V., Moreau R., Jalan R., Ginès P. (2015). EASL-CLIF Consortium CANONIC Study Acute-on-Chronic Liver Failure: A New Syndrome That Will Re-Classify Cirrhosis. J. Hepatol..

[84] Xie F., Yan L., Lu J., Zheng T., Shi C., Ying J., Shen R., Yang J. (2013). Effects of Nucleoside Analogue on Patients with Chronic Hepatitis B-Associated Liver Failure: Meta-Analysis. PLoS ONE.

[85] Bajaj J.S., O’Leary J.G., Reddy K.R., Wong F., Biggins S.W., Patton H., Fallon M.B., Garcia-Tsao G., Maliakkal B., Malik R. (2014). Survival in Infection-Related Acute-on-Chronic Liver Failure Is Defined by Extrahepatic Organ Failures. Hepatology.

[86] Sargenti K., Prytz H., Nilsson E., Bertilsson S., Kalaitzakis E. (2015). Bacterial Infections in Alcoholic and Nonalcoholic Liver Cirrhosis. Eur. J. Gastroenterol. Hepatol..

[87] Solà E., Solé C., Simón-Talero M., Martín-Llahí M., Castellote J., Garcia-Martínez R., Moreira R., Torrens M., Márquez F., Fabrellas N. (2018). Midodrine and Albumin for Prevention of Complications in Patients with Cirrhosis Awaiting Liver Transplantation. A Randomized Placebo-Controlled Trial. J. Hepatol..

[88] O’Brien A., Kamath P.S., Trotter J. (2018). MACHT—Outpatient Albumin Infusions Do Not Prevent Complications of Cirrhosis in Patients on the Liver Transplant Waiting List. J. Hepatol..

[89] Caraceni P., Riggio O., Angeli P., Alessandria C., Neri S., Foschi F.G., Levantesi F., Airoldi A., Boccia S., Svegliati-Baroni G. (2018). Long-Term Albumin Administration in Decompensated Cirrhosis (ANSWER): An Open-Label Randomised Trial. Lancet.

[90] China L., Freemantle N., Forrest E., Kallis Y., Ryder S.D., Wright G., Portal A.J., Becares Salles N., Gilroy D.W., O’Brien A. (2021). A Randomized Trial of Albumin Infusions in Hospitalized Patients with Cirrhosis. N. Engl. J. Med..

[91] China L., Skene S.S., Shabir Z., Maini A., Sylvestre Y., Bennett K., Bevan S., O’Beirne J., Forrest E., Portal J. (2018). Administration of Albumin Solution Increases Serum Levels of Albumin in Patients With Chronic Liver Failure in a Single-Arm Feasibility Trial. Clin. Gastroenterol. Hepatol..

[92] Siddiqui M.S., Stravitz R.T. (2014). Intensive Care Unit Management of Patients with Liver Failure. Clin. Liver Dis..

[93] Olson J.C., Wendon J.A., Kramer D.J., Arroyo V., Jalan R., Garcia-Tsao G., Kamath P.S. (2011). Intensive Care of the Patient with Cirrhosis. Hepatology.

[94] Lai J.C., Tandon P., Bernal W., Tapper E.B., Ekong U., Dasarathy S., Carey E.J. (2021). Malnutrition, Frailty, and Sarcopenia in Patients With Cirrhosis: 2021 Practice Guidance by the American Association for the Study of Liver Diseases. Hepatology.

[95] Plauth M., Bernal W., Dasarathy S., Merli M., Plank L.D., Schütz T., Bischoff S.C. (2019). ESPEN Guideline on Clinical Nutrition in Liver Disease. Clin. Nutr..

[96] European Association for the Study of the Liver (2019). Electronic address: Easloffice@easloffice.eu; European Association for the Study of the Liver EASL Clinical Practice Guidelines on Nutrition in Chronic Liver Disease. J. Hepatol..

[97] Moreno C., Deltenre P., Senterre C., Louvet A., Gustot T., Bastens B., Hittelet A., Piquet M.-A., Laleman W., Orlent H. (2016). Intensive Enteral Nutrition Is Ineffective for Patients With Severe Alcoholic Hepatitis Treated With Corticosteroids. Gastroenterology.

[98] Yang J., Sun H., Liu Q. (2016). The Comparative Efficacy and Safety of Entecavir and Lamivudine in Patients with HBV-Associated Acute-on-Chronic Liver Failure: A Systematic Review and Meta-Analysis. Gastroenterol. Res. Pract..

[99] Li J., Hu C., Chen Y., Zhang R., Fu S., Zhou M., Gao Z., Fu M., Yan T., Yang Y. (2021). Short-Term and Long-Term Safety and Efficacy of Tenofovir Alafenamide, Tenofovir Disoproxil Fumarate and Entecavir Treatment of Acute-on-Chronic Liver Failure Associated with Hepatitis B. BMC Infect. Dis..

[100] Yang J., Chen G., Chen X., Zhang H., Jiang D., Yang G. (2015). Initial Combination Anti-Viral Therapy with Lamivudine and Adefovir Dipivoxil Decreases Short-Term Fatality Rate of Hepatitis-B-Virus-Related Acute-on-Chronic Liver Failure. Virol. J..

[101] Sarin S.K., Kumar M., Lau G.K., Abbas Z., Chan H.L.Y., Chen C.J., Chen D.S., Chen H.L., Chen P.J., Chien R.N. (2016). Asian-Pacific Clinical Practice Guidelines on the Management of Hepatitis B: A 2015 Update. Hepatol. Int..

[102] Wu T., Li J., Shao L., Xin J., Jiang L., Zhou Q., Shi D., Jiang J., Sun S., Jin L. (2018). Development of Diagnostic Criteria and a Prognostic Score for Hepatitis B Virus-Related Acute-on-Chronic Liver Failure. Gut.

[103] Zhao R.-H., Shi Y., Zhao H., Wu W., Sheng J.-F. (2018). Acute-on-Chronic Liver Failure in Chronic Hepatitis B: An Update. Expert Rev. Gastroenterol. Hepatol..

[104] Laursen T.L., Sandahl T.D., Kazankov K., George J., Grønbæk H. (2020). Liver-Related Effects of Chronic Hepatitis C Antiviral Treatment. World J. Gastroenterol..

[105] Poordad F., Hezode C., Trinh R., Kowdley K.V., Zeuzem S., Agarwal K., Shiffman M.L., Wedemeyer H., Berg T., Yoshida E.M. (2014). ABT-450/r-Ombitasvir and Dasabuvir with Ribavirin for Hepatitis C with Cirrhosis. N. Engl. J. Med..

[106] Poordad F., Schiff E.R., Vierling J.M., Landis C., Fontana R.J., Yang R., McPhee F., Hughes E.A., Noviello S., Swenson E.S. (2016). Daclatasvir with Sofosbuvir and Ribavirin for Hepatitis C Virus Infection with Advanced Cirrhosis or Post-Liver Transplantation Recurrence. Hepatology.

[107] Pungpapong S., Aqel B., Leise M., Werner K.T., Murphy J.L., Henry T.M., Ryland K., Chervenak A.E., Watt K.D., Vargas H.E. (2015). Multicenter Experience Using Simeprevir and Sofosbuvir with or without Ribavirin to Treat Hepatitis C Genotype 1 after Liver Transplant. Hepatology.

[108] Ferenci P., Bernstein D., Lalezari J., Cohen D., Luo Y., Cooper C., Tam E., Marinho R.T., Tsai N., Nyberg A. (2014). ABT-450/r-Ombitasvir and Dasabuvir with or without Ribavirin for HCV. N. Engl. J. Med..

[109] Mandorfer M., Kozbial K., Schwabl P., Freissmuth C., Schwarzer R., Stern R., Chromy D., Stättermayer A.F., Reiberger T., Beinhardt S. (2016). Sustained Virologic Response to Interferon-Free Therapies Ameliorates HCV-Induced Portal Hypertension. J. Hepatol..

[110] Mauro E., Crespo G., Montironi C., Londoño M.-C., Hernández-Gea V., Ruiz P., Sastre L., Lombardo J., Mariño Z., Díaz A. (2018). Portal Pressure and Liver Stiffness Measurements in the Prediction of Fibrosis Regression after Sustained Virological Response in Recurrent Hepatitis C. Hepatology.

[111] Kamar N., Izopet J., Tripon S., Bismuth M., Hillaire S., Dumortier J., Radenne S., Coilly A., Garrigue V., D’Alteroche L. (2014). Ribavirin for Chronic Hepatitis E Virus Infection in Transplant Recipients. N. Engl. J. Med..

[112] Goyal R., Kumar A., Panda S.K., Paul S.B., Acharya S.K. (2012). Ribavirin Therapy for Hepatitis E Virus-Induced Acute on Chronic Liver Failure: A Preliminary Report. Antivir. Ther..

[113] Pischke S., Hardtke S., Bode U., Birkner S., Chatzikyrkou C., Kauffmann W., Bara C.L., Gottlieb J., Wenzel J., Manns M.P. (2013). Ribavirin Treatment of Acute and Chronic Hepatitis E: A Single-Centre Experience. Liver Int..

[114] Arroyo V., Moreau R., Jalan R. (2020). Acute-on-Chronic Liver Failure. N. Engl. J. Med..

[115] Singer M., Deutschman C.S., Seymour C.W., Shankar-Hari M., Annane D., Bauer M., Bellomo R., Bernard G.R., Chiche J.-D., Coopersmith C.M. (2016). The Third International Consensus Definitions for Sepsis and Septic Shock (Sepsis-3). JAMA.

[116] Tapper E.B., Parikh N.D., Sengupta N., Mellinger J., Ratz D., Lok A.S.-F., Su G.L. (2018). A Risk Score to Predict the Development of Hepatic Encephalopathy in a Population-Based Cohort of Patients with Cirrhosis. Hepatology.

[117] Bajaj J.S., O’Leary J.G., Tandon P., Wong F., Garcia-Tsao G., Kamath P.S., Maliakkal B., Biggins S.W., Thuluvath P.J., Fallon M.B. (2017). Hepatic Encephalopathy Is Associated With Mortality in Patients With Cirrhosis Independent of Other Extrahepatic Organ Failures. Clin. Gastroenterol. Hepatol..

[118] Piscaglia A.C., Arena V., Passalacqua S., Gasbarrini A. (2015). A Case of Granulocyte-Colony Stimulating Factor/Plasmapheresis-Induced Activation of Granulocyte-Colony Stimulating Factor-Positive Hepatic Progenitors in Acute-on-Chronic Liver Failure. Hepatology.

[119] Gustot T. (2014). Beneficial Role of G-CSF in Acute-on-Chronic Liver Failure: Effects on Liver Regeneration, Inflammation/Immunoparalysis or Both?. Liver Int..

[120] Pittenger M.F., Mackay A.M., Beck S.C., Jaiswal R.K., Douglas R., Mosca J.D., Moorman M.A., Simonetti D.W., Craig S., Marshak D.R. (1999). Multilineage Potential of Adult Human Mesenchymal Stem Cells. Science.

[121] Xue R., Meng Q., Dong J., Li J., Yao Q., Zhu Y., Yu H. (2018). Clinical Performance of Stem Cell Therapy in Patients with Acute-on-Chronic Liver Failure: A Systematic Review and Meta-Analysis. J. Transl. Med..

[122] Engelmann C., Sheikh M., Sharma S., Kondo T., Loeffler-Wirth H., Zheng Y.B., Novelli S., Hall A., Kerbert A.J.C., Macnaughtan J. (2020). Toll-like Receptor 4 Is a Therapeutic Target for Prevention and Treatment of Liver Failure. J. Hepatol..

[123] Bhattacharyya S., Wang W., Tamaki Z., Shi B., Yeldandi A., Tsukimi Y., Yamasaki M., Varga J. (2018). Pharmacological Inhibition of Toll-Like Receptor-4 Signaling by TAK242 Prevents and Induces Regression of Experimental Organ Fibrosis. Front. Immunol..

[124] Matsunaga N., Tsuchimori N., Matsumoto T., Ii M. (2011). TAK-242 (Resatorvid), a Small-Molecule Inhibitor of Toll-like Receptor (TLR) 4 Signaling, Binds Selectively to TLR4 and Interferes with Interactions between TLR4 and Its Adaptor Molecules. Mol. Pharmacol..

[125] Klein J., Ernst M., Christmann A., Tropper M., Leykauf T., Kreis W., Munkert J. (2021). Knockout of Arabidopsis Thaliana VEP1, Encoding a PRISE (Progesterone 5β-Reductase/Iridoid Synthase-Like Enzyme), Leads to Metabolic Changes in Response to Exogenous Methyl Vinyl Ketone (MVK). Metabolites.

[126] Peters E., Masereeuw R., Pickkers P. (2014). The Potential of Alkaline Phosphatase as a Treatment for Sepsis-Associated Acute Kidney Injury. Nephron Clin. Pract..

[127] Gustot T., Jalan R. (2019). Acute-on-Chronic Liver Failure in Patients with Alcohol-Related Liver Disease. J. Hepatol..

[128] Hall A.R., Burke N., Dongworth R.K., Hausenloy D.J. (2014). Mitochondrial Fusion and Fission Proteins: Novel Therapeutic Targets for Combating Cardiovascular Disease. Br. J. Pharmacol..

[129] Xue R., Yang J., Jia L., Zhu X., Wu J., Zhu Y., Meng Q. (2019). Mitofusin2, as a Protective Target in the Liver, Controls the Balance of Apoptosis and Autophagy in Acute-on-Chronic Liver Failure. Front. Pharmacol..

[130] Bosch J., Gracia-Sancho J., Abraldes J.G. (2020). Cirrhosis as New Indication for Statins. Gut.

[131] Trebicka J., Hennenberg M., Laleman W., Shelest N., Biecker E., Schepke M., Nevens F., Sauerbruch T., Heller J. (2007). Atorvastatin Lowers Portal Pressure in Cirrhotic Rats by Inhibition of RhoA/Rho-Kinase and Activation of Endothelial Nitric Oxide Synthase. Hepatology.

[132] Marrone G., Maeso-Díaz R., García-Cardena G., Abraldes J.G., García-Pagán J.C., Bosch J., Gracia-Sancho J. (2015). KLF2 Exerts Antifibrotic and Vasoprotective Effects in Cirrhotic Rat Livers: Behind the Molecular Mechanisms of Statins. Gut.

[133] Pollo-Flores P., Soldan M., Santos U.C., Kunz D.G., Mattos D.E., da Silva A.C., Marchiori R.C., Rezende G.F. (2015). da M. Three Months of Simvastatin Therapy vs. Placebo for Severe Portal Hypertension in Cirrhosis: A Randomized Controlled Trial. Dig. Liver Dis..

[134] Millea P.J. (2009). N-Acetylcysteine: Multiple Clinical Applications. Am. Fam. Physician.

[135] Otrubová O., Turecký L., Uličná O., Janega P., Luha J., Muchová J. (2018). Therapeutic Effects of N-Acetyl-L-Cysteine on Liver Damage Induced by Long-Term CCl4 Administration. Gen. Physiol. Biophys..

[136] Wang M.-L., Yin X.-J., Li X.-L., Wang F.-D., Zhou J., Tao Y.-C., Wang Y.-H., Wu D.-B., Chen E.-Q. (2021). Retrospective Analysis of the Clinical Efficacy of N-Acetylcysteine in the Treatment of Hepatitis B Virus Related Acute-on-Chronic Liver Failure. Front. Med..

[137] Khanam A., Kottilil S. (2021). Acute-on-Chronic Liver Failure: Pathophysiological Mechanisms and Management. Front. Med..

[138] Chan A.C.Y., Fan S.T. (2015). Criteria for Liver Transplantation in ACLF and Outcome. Hepatol. Int..

[139] Sundaram V., Jalan R., Wu T., Volk M.L., Asrani S.K., Klein A.S., Wong R.J. (2019). Factors Associated with Survival of Patients With Severe Acute-On-Chronic Liver Failure Before and After Liver Transplantation. Gastroenterology.

[140] Abbas N., Rajoriya N., Elsharkawy A.M., Chauhan A. (2022). Acute-on-Chronic Liver Failure (ACLF) in 2022: Have Novel Treatment Paradigms Already Arrived?. Expert Rev. Gastroenterol. Hepatol..

[141] Goldberg D.S., Bajaj J.S. (2022). Acute-on-Chronic Liver Failure and Liver Transplantation: Putting the Cart Before the Horse in Data Analyses and Advocating for Model for End-Stage Liver Disease Exceptions. Liver Transpl..

[142] Abdallah M.A., Waleed M., Bell M.G., Nelson M., Wong R., Sundaram V., Singal A.K. (2020). Systematic Review with Meta-Analysis: Liver Transplant Provides Survival Benefit in Patients with Acute on Chronic Liver Failure. Aliment. Pharmacol. Ther..

[143] Karvellas C.J., Subramanian R.M. (2016). Current Evidence for Extracorporeal Liver Support Systems in Acute Liver Failure and Acute-on-Chronic Liver Failure. Crit. Care Clin..

[144] Kribben A., Gerken G., Haag S., Herget-Rosenthal S., Treichel U., Betz C., Sarrazin C., Hoste E., Van Vlierberghe H., Escorsell A. (2012). Effects of Fractionated Plasma Separation and Adsorption on Survival in Patients with Acute-on-Chronic Liver Failure. Gastroenterology.

[145] Bañares R., Nevens F., Larsen F.S., Jalan R., Albillos A., Dollinger M., Saliba F., Sauerbruch T., Klammt S., Ockenga J. (2013). Extracorporeal Albumin Dialysis with the Molecular Adsorbent Recirculating System in Acute-on-Chronic Liver Failure: The RELIEF Trial. Hepatology.

[146] Yue-Meng W., Yang L.-H., Yang J.-H., Xu Y., Yang J., Song G.-B. (2016). The Effect of Plasma Exchange on Entecavir-Treated Chronic Hepatitis B Patients with Hepatic de-Compensation and Acute-on-Chronic Liver Failure. Hepatol. Int..

[147] Jalan R., Pavesi M., Saliba F., Amorós A., Fernandez J., Holland-Fischer P., Sawhney R., Mookerjee R., Caraceni P., Moreau R. (2015). The CLIF Consortium Acute Decompensation Score (CLIF-C ADs) for Prognosis of Hospitalised Cirrhotic Patients without Acute-on-Chronic Liver Failure. J. Hepatol..

[148] Thanapirom K., Teerasarntipan T., Treeprasertsuk S., Choudhury A., Sahu M.K., Maiwall R., Pamecha V., Moreau R., Al Mahtab M., Chawla Y.K. (2022). Impact of Compensated Cirrhosis on Survival in Patients with Acute-on-Chronic Liver Failure. Hepatol. Int..

[149] Shalimar, Kumar D., Vadiraja P.K., Nayak B., Thakur B., Das P., Gupta S.D., Panda S.K., Acharya S.K. (2016). Acute on Chronic Liver Failure Because of Acute Hepatic Insults: Etiologies, Course, Extrahepatic Organ Failure and Predictors of Mortality. J. Gastroenterol. Hepatol..

[150] Shin J., Yu J.H., Jin Y.-J., Yim H.J., Jung Y.K., Yang J.M., Song D.S., Kim Y.S., Kim S.G., Kim D.J. (2020). Acute-on-Chronic Liver Failure as a Major Predictive Factor for Mortality in Patients with Variceal Bleeding. Clin. Mol. Hepatol..

[151] Shalimar, Saraswat V., Singh S.P., Duseja A., Shukla A., Eapen C.E., Kumar D., Pandey G., Venkataraman J., Puri P. (2016). Acute-on-Chronic Liver Failure in India: The Indian National Association for Study of the Liver Consortium Experience. J. Gastroenterol. Hepatol..

[152] Karvellas C.J., Bagshaw S.M. (2014). Advances in Management and Prognostication in Critically Ill Cirrhotic Patients. Curr. Opin. Crit. Care.

[153] Mikolasevic I., Milic S., Radic M., Orlic L., Bagic Z., Stimac D. (2015). Clinical Profile, Natural History, and Predictors of Mortality in Patients with Acute-on-Chronic Liver Failure (ACLF). Wien. Klin. Wochenschr..

[154] Garg H., Kumar A., Garg V., Sharma P., Sharma B.C., Sarin S.K. (2012). Clinical Profile and Predictors of Mortality in Patients of Acute-on-Chronic Liver Failure. Dig. Liver Dis..

